# Exosome-mediated effects and applications in inflammatory diseases of the digestive system

**DOI:** 10.1186/s40001-022-00792-y

**Published:** 2022-08-31

**Authors:** Xianli Wu, Xiaolin Xu, Yiwei Xiang, Dongdong Fan, Qiming An, Gengyu Yue, Zhe Jin, Jianhong Ding, Yanxia Hu, Qian Du, Jingyu Xu, Rui Xie

**Affiliations:** 1 Department of Gastroenterology, Digestive Disease Hospital, Affiliated Hospital of Zunyi Medical University, Zunyi, China; 2 The Collaborative Innovation Center of Tissue Damage Repair and Regeneration Medicine of Zunyi Medical University, Zunyi, China

**Keywords:** Exosomes, Esophagitis, Gastritis, Inflammatory bowel disease Hepatitis, Pancreatitis

## Abstract

Exosomes are membranous vesicles containing RNA and proteins that are specifically secreted in vivo. Exosomes have many functions, such as material transport and signal transduction between cells. Many studies have proven that exosomes can not only be used as biomarkers for disease diagnosis but also as carriers to transmit information between cells. Exosomes participate in a variety of physiological and pathological processes, including the immune response, antigen presentation, cell migration, cell differentiation, and tumour development. Differences in exosome functions depend on cell type. In recent years, exosome origin, cargo composition, and precise regulatory mechanisms have been the focus of research. Although exosomes have been extensively reported in digestive tumours, few articles have reviewed their roles in inflammatory diseases of the digestive system, especially inflammatory-related diseases (such as reflux oesophagitis, gastritis, inflammatory bowel disease, hepatitis, and pancreatitis). This paper briefly summarizes the roles of exosomes in inflammatory diseases of the digestive system to provide a basis for research on the mechanism of inflammatory diseases of the digestive system targeted by exosomes.

## Preface

The digestive system is one of the most important systems in the human body and provides materials and energy. Because of the modern fast-paced lifestyle, digestive system diseases occur frequently. The incidence of digestive system tumours is also gradually increasing, causing serious social and economic burdens. In recent years, people have paid increasing attention to inflammatory diseases of the digestive system. On the one hand, chronic inflammation is an important factor in tumour development. The inflammatory microenvironment can increase the probability of cellular mutation and the proliferative capacity of mutant cells. Inflammation can activate endogenous or exogenous signals involving a variety of proteins and inflammatory mediators by changing the environment of tumour cells [1, 2] and promote the occurrence and development of cancer. For example, chronic atrophic gastritis (CAG) can lead to intestinal metaplasia and atypical hyperplasia and further carcinogenesis [3]. Long-term chronic inflammation of the colon and rectum may also lead to cancer development [4]. Pancreatic cancer [5] and liver cancer [6] have also been shown to be strongly associated with chronic inflammation. On the other hand, digestive system inflammatory disease easily recurs and is not easily cured, seriously affecting quality of life. Therefore, studying inflammatory diseases of the digestive system is necessary. Recently, research on exosomes has been growing. Some scholars have examined the relationship between exosomes and inflammatory diseases of the digestive system, which has provided new breakthroughs for the diagnosis and treatment of digestive inflammatory diseases.

## Introduction to exosomes

### Secretion and composition of exosomes

**Fig. 1:**
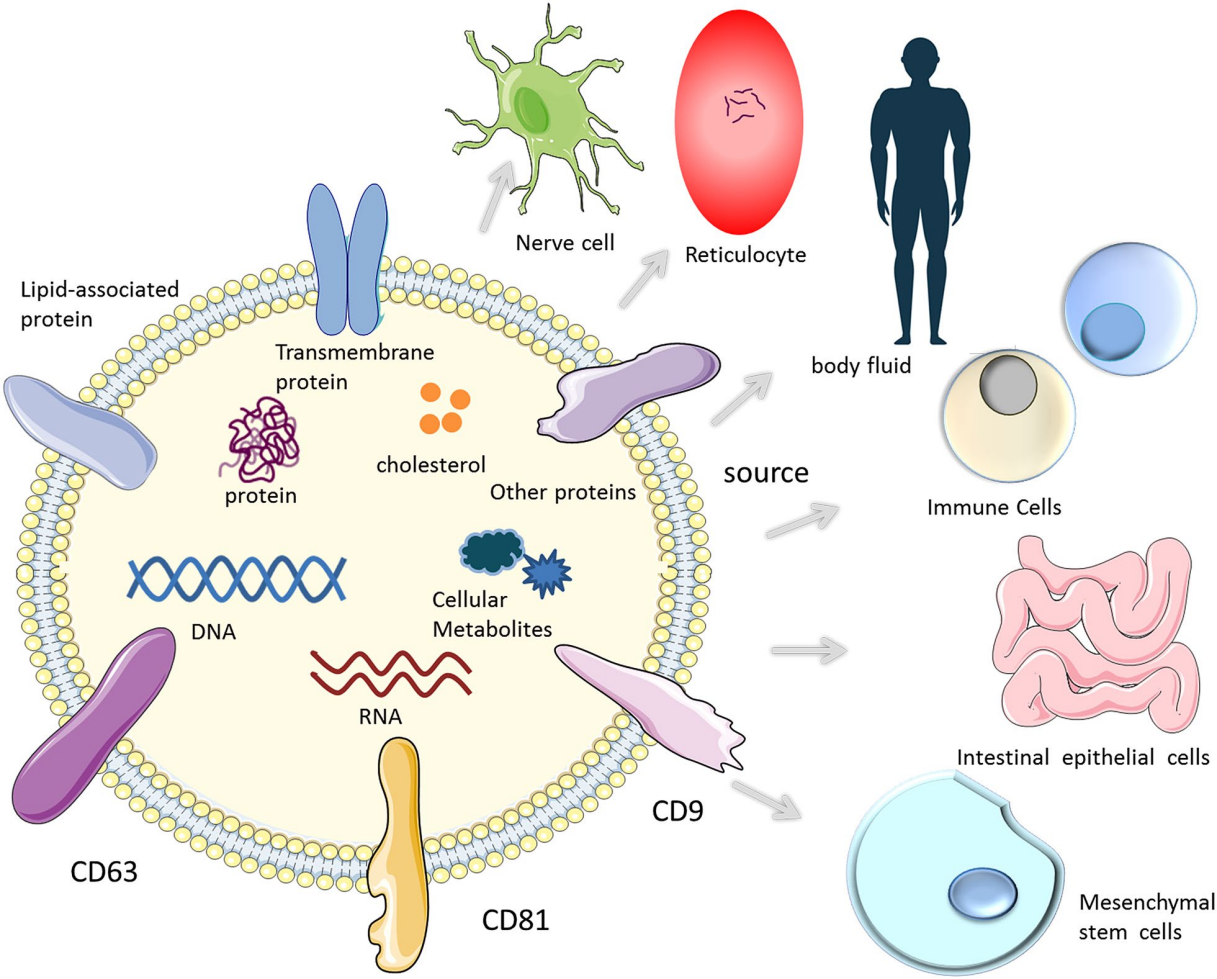
Composition and sources of exosomes. Exosomes are mainly composed of lipids, nucleic acids and proteins. Its outer membrane is a lipid bilayer structure and contains specific biological substances, mainly including tetraspanins, lipid-related proteins, and membrane transporters. Exosomes are widely sourced and present in most cells and body fluids (such as immune cells, mesenchymal stem cells, reticulocytes, nerve cells, urine, saliva and blood)

**Fig. 2:**
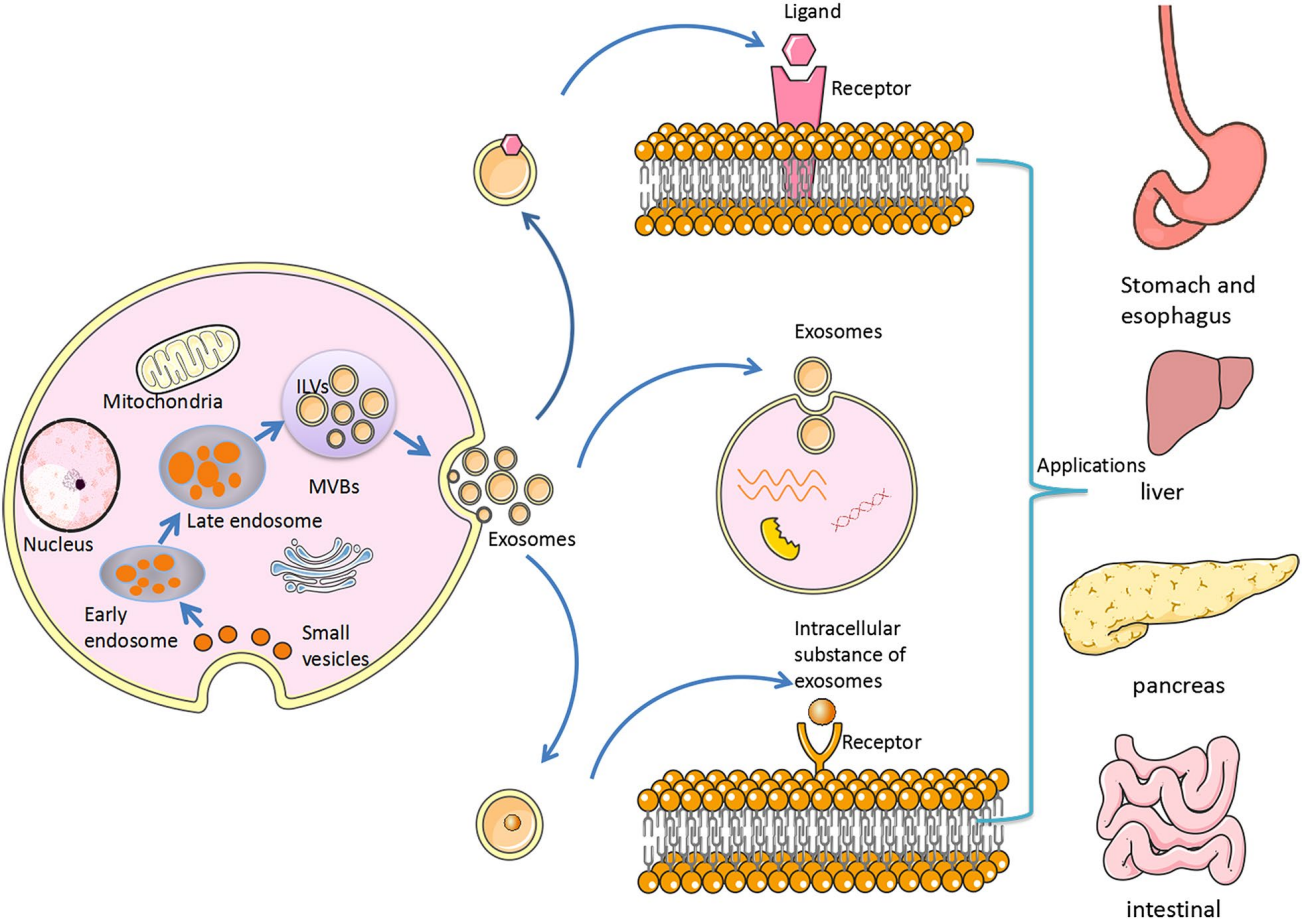
Secretion and application of exosomes. Release of exosomes: the plasma membrane is invaginated and some extracellular components and cell membrane proteins are wrapped together to form early endosomes. These early endosomes can exchange substances with other organelles, or these different early endosomes fuse with each other to form late endosomes. and further form intracellular multivesicular bodies, which will contain many intraluminal vesicles. These ILVs may be released in the future to become exosomes. These vesicles can act on various organs in the body (such as the oesophagus, stomach, intestine, liver, and pancreas) to exert their biological effects

Exosomes belong to a subgroup of extracellular vesicles, which are tiny membrane vesicles that can be secreted by most cells in the body, with a lipid bilayer membrane, about 30–150 nm in diameter, and were first discovered in reticulocytes [7–9]. The process of exosome production is as follows: the plasma membrane is invaginated and some extracellular components and cell membrane proteins are wrapped together to form early endosomes. These early endosomes can exchange substances with other organelles, or these different early endosomes fuse with each other to form late endosomes and further form intracellular multivesicular bodies, which will contain many intraluminal vesicles. These ILVs may be released in the future to become exosomes. After the cells form MVBs, they may be degraded by fusion with autophagosomes or lysosomes, or they may be fused with the plasma membrane to release the substances in them, including ILVs, which are the final exosomes [10].Exosomes can be secreted by most cells, such as B cells [11], T cells [12], mast cells [13], mesenchymal stem cells (MSCs) [14], reticulocytes [7] and nerve cells [15], under normal or pathological conditions. Exosomes are also widely found in urine, saliva, blood, and milk [16–18]. Studies have shown that exosomes contain specific biological substances, mainly lipids, nucleic acids and proteins [19]. Lipids components mainly include phosphatidylserine, sphingomyelin, cholesterol, arachidonic acid and prostaglandins, etc. Common proteins include membrane transport proteins and fusion proteins, tetraspanins (CD9, CD63, CD81, CD82), chaperones, polycystic endosomal synthesis proteins, cytoskeletal proteins, and lipoproteins [20]. In addition, DNA, mRNA, microRNAs and intermediate metabolites are also involved [21, 22]. These substances not only reflect the original cell types but, more importantly, are closely related to the physiological function of or pathological changes in the original cells (Figs. 1 and 2).

### Function of exosomes

Exosomes are derived from a variety of cell types, and the differences in their functions depends on the cell source. The exosome membrane is composed of a phospholipid bilayer and some functional proteins. The main contents include proteins, nucleic acids, and lipids, which participate in exosomal functions. The unique lipid bilayer structure of exosomes enables them to stably exist in interstitial fluid. This special structure can not only protect exosomes from degradation but also ensures that the RNA and proteins inside exosomes, which are easily degraded, are protected from damage. The specific membrane proteins on exosomes and their contents can be used as biomarkers for disease diagnosis. Exosomes can transfer corresponding biomolecules to participate in the occurrence and development of various diseases and act on various biological processes, such as the inflammatory response, the immune response, apoptosis, and intercellular signal transduction [11, 23, 24].It has been found that when TECs (tubular epithelial cells) are treated with BSA (bovine serum albumin), the production of exosomes in the supernatant increases. Further studies found that CCL2 mRNA was selectively enriched in the TEC exosomes after BSA stimulation. CCL2 mRNA can be delivered to macrophages via exosomes, leading to macrophage activation and migration, ultimately promoting tubulointerstitial inflammation [25]. Another study have shown that MSC-derived exosomes are involved in inhibiting cell cycle progression in cell lines and inducing apoptosis in HepG2 cells and Kaposi cells [26]. Studies related to exosomes and viruses have shown that exosomes can transmit viral proteins [27] and microRNAs [27] and carry RNA viral components to promote virus infection between tissue cells. Some non-enveloped RNA viruses rely on the pathway of exosome generation, using the bilayer membrane of exosomes as an acquired capsule to resist neutralization by host antibodies and escape host immunity [28]. In addition, studies have shown that exosomes exert their effects mainly by interacting with receptor cells [29], and they can play their biological roles in a variety of ways. First, exosomes can transmit cellular information by the binding of ligands on their membrane with receptors on the target cell membrane. Second, exosomes can fuse with target cells and release factors and nucleic acids to carry out information transmission. In addition, exosomes can release intracellular substances, which act on receptors on the surface of target cells, transmit information transmission and induce biological effects (Fig. 2). In conclusion, exosomes play an important role in all aspects of biological life. The rich biological information carried in exosomes can play an important roles in improving the cell microenvironment, participating in immune regulation, mediating cell communication and other biological functions, and participating in the occurrence and development of diseases.

### Current status of exosomes in inflammatory diseases

Inflammatory disease is common in various organs. With increasing research on exosomes, their role in inflammatory disease has been gradually elucidated. Studies have shown exosomes in the upper respiratory tract [30]. Nasal exosomes can participate in cell-to-cell communication among immune cells in the upper respiratory tract, and their protein cargoes are involved in immune regulation. In subjects with airway disease, the expression of nasal exosomal mucin and serum-related proteins was increased, while the expression of barrier proteins and antimicrobial proteins was decreased, suggesting that nasal exosomal proteins play an important role in disease progression [31]. Studies suggest that eosinophils can secrete exosomes, while eosinophils from asthma patients release more exosomes than those from healthy individuals. Exosomes derived from eosinophils induce the production of reactive oxygen species (ROS) and nitric oxide (NO) in eosinophils. In addition, exosomes may contain chemokines produced by eosinophils, increasing cell adhesion and leading to an increase in the specificity of adhesion molecules (such as ICAM-1 and integrin α2), which are involved in driving the development and maintenance of asthma [32, 33]. In addition, studies have shown [32] that exosomes can be used as ideal drug delivery vehicles for neuroinflammatory diseases, transporting drugs through the blood–brain barrier and thereby delaying the progression of encephalitis. A study examined the delivery of curcumin encapsulated by exosomes from the nasal cavity to the brain to treat encephalitis, and it was found that curcumin encapsulated by exosomes can be rapidly transported to the brain to induce apoptosis in activated microglia, delaying the progression of experimental autoimmune encephalitis in rats [34]. The study also identified exosomal in the urine of patients with lupus nephritis, and expression increased significantly during active disease. In particular, exosomal miR-146a in urine was positively correlated with the disease activity of lupus nephritis. Therefore, miR-146a in urinary exosomes may be a reference for the evaluation of lupus nephritis [35]. In studies of exosomes released by cells infected with mycobacteria, macrophages infected with mycobacteria released more exosomes and expressed more Hsp70 on their surfaces, activating NF-κB and stimulating the release of tumour necrosis factor alpha (TNFα), leading to proinflammatory responses [36]. In conclusion, exosomes are increasingly used in inflammatory diseases and play important roles in pathogenesis, diagnosis and treatment.

## Exosomes and digestive system inflammatory diseases

### Exosomes and reflux oesophagitis

Reflux oesophagitis (RE) is an inflammatory disease of the oesophagus caused by movement of stomach and duodenum contents back into the oesophagus and is a gastroesophageal reflux disease (GERD). At present, RE diagnosis is mainly based on symptoms and gastroscopy. Treatment is mainly the use of proton pump inhibitors, and patients are prone to recurrent attacks, which increases the incidence of oesophageal adenocarcinoma. Many studies have shown that exosomal miRNAs may act as biomarkers for the diagnosis and prognosis of oesophageal cancer. Tanaka et al. found that serum exosomal miR-21 was upregulated in patients with oesophageal squamous cell carcinoma (ESCC), and the expression level of exosomal miR-21 was positively correlated with tumour progression and prognosis; thus, exosomal miR-21 may be a promising target for experimental ESCC therapy [37]. Studies have also shown that miR-375 overexpression can inhibit the migration and invasion of ESCC cells [38]. Enamelin (ENAH) was identified as the target gene of miR-375. Exosomal miR-375 derived from human umbilical cord MSCs (hucMSCs) inhibited the expression of ENAH and the proliferation, invasion, and migration of ESCC cells [39]. In addition, the role of exosomes in RE has been reported in recent years. A study examined the expression of serum exosomal miRNA in rats with RE, and it was found that serum exosomal miR-29a-3p levels were significantly increased in rats with chronic RE but not in rats with gastric ulcer or colitis, suggesting that serum exosomal miR-29a-3p may be a specific marker of chronic RE [40](Table 1). Yan et al. reported that the expression of miR-203 in exosomes from GERD patients was significantly downregulated, suggesting that detection of miR-203 in tongue coating samples may contribute to the diagnosis of GERD [41]. These studies suggest that exosomal miRNAs can be used as markers and to diagnose GERD.

### Exosomes and chronic gastritis

Chronic gastritis is a common digestive system disease that involves chronic inflammation of the gastric mucosa caused by various aetiological factors. The clinical manifestations can vary. Diagnosis mainly depends on gastroscopy and pathological biopsy of the gastric mucosa. If chronic gastritis is not diagnosed and treated in time, it easily develops into gastric ulcers and gastric cancer, seriously affecting quality of life. Many studies have shown that exosomes play an important role in gastrointestinal tumours, and the role of exosomes in chronic gastritis has been gradually explored.

Helicobacter pylori (HP) infection is one of the common causes of chronic gastritis. It has been reported that exosomes can affect inflammatory cytokines and play a proinflammatory role in the pathogenesis of HP-positive chronic gastritis. Chen et al. found that serum exosomes from HP-positive chronic gastritis patients can be absorbed by gastric epithelial cells. The expression of the proinflammatory cytokine IL-1α is regulated by IL-6/sIL-6R trans-signalling, which promotes the inflammatory response [42]. In addition, studies have shown that [43] HP infection can stimulate miR-155 expression in gastric epithelial cells and gastric mucosal tissues, and miR-155 in exosomes produced by HP-infected macrophages was also significantly upregulated. Therefore, miR-155 may regulate inflammation in host cells through exosomes produced by different immune cells. Some studies have reported [44] that exosomes deliver miR-155 to macrophages, regulate the expression of various proinflammatory mediators and inflammation-related proteins in macrophages, and increase the expression of the cytokines TNF-α, IL-6, and IL-23 and the signal transduction proteins CD40, CD63, CD81 and MHC-I in macrophages. However, the expression of MyD88 and NF-κB was downregulated, suggesting that exosomal miR-155 was involved in regulating the inflammatory response to HP infection and then regulated the occurrence and development of gastrointestinal diseases.

Many studies have shown that exosomal miRNAs are involved in the regulation of many diseases. Because of their high stability and disease-specific and tissue-specific expression profiles, miRNAs are considered a new class of valuable biomarkers by many researchers. The diagnostic methods of many tumour diseases are complex, time consuming and mostly invasive. Therefore, to find a simple way to diagnose diseases, many researchers have studied exosomal miRNAs as biomarkers in various tumour diseases and have made a series of achievements. For example, it has been reported that miR-19b-3p and miR-106a-5p were overexpressed in patients with gastric cancer and could be used as biomarkers of gastric cancer [45]. Plasma exosomal miR-485-3p and miR-4433a-5p were identified as biomarkers for the diagnosis of papillary thyroid carcinoma (PTC), and plasma exosomal miR-485-3p can be used to distinguish between high-risk and low-risk PTC [46]. In addition, in recent years, with the increasing prevalence of digestive diseases, the study of exosomal miRNAs has been focused on chronic gastritis. In a study of the mechanism of action of miRNAs in serum exosomes of patients with CAG [47], the expression of hsa-miR-122-5p in serum exosomes was significantly upregulated in patients with CAG compared to those with chronic non-atrophic gastritis. Hsa-miR-122-5p in serum exosomes may serve as a potential biomarker for the diagnosis of CAG, indicating that miRNAs in exosomes can be used to diagnose CAG.

### Exosomes and inflammatory bowel disease

Inflammatory bowel disease (IBD) is a chronic nonspecific intestinal inflammatory disease that includes ulcerative colitis (UC), Crohn's disease (CD), and unshaped colitis. Its aetiology and pathogenesis are not fully understood. Recent studies have shown that genetic and environmental factors, immune response abnormalities, intestinal barrier dysfunction and intestinal flora imbalances are related to the development of IBD. The clinical manifestations are chronic abdominal pain, diarrhoea, and easily repeated mucus empyema [48]. Treatments mainly rely on anti-inflammatory drugs, immunosuppressive agents, purine drugs and anti-TNF monoclonal antibodies, but the therapeutic efficacy is still not ideal. Studies have shown that exosomes are involved in IBD pathogenesis and play an important role in diagnosis and treatment.

Several studies have shown that IBD pathogenesis is related to intestinal mucosal innate immunity and acquired immunodeficiency or abnormal responses [49–51]. The imbalance between intestinal anti-inflammatory and inflammatory factors is an important mechanism of IBD pathogenesis. In the intestinal mucosa, inflammatory cell infiltration can lead to intestinal lesions. Macrophages play an important role in the inflammatory response. Macrophage activation is essential for IBD pathogenesis [52], and exosomes may alleviate colon tissue damage and inhibit further development of IBD by inhibiting macrophage functions. In animal experiments to investigate the therapeutic effects of exosomes released by hucMSCs on dextran sulfate sodium (DSS)-induced IBD, exosomes derived from hucMSCs reduced the number of murine macrophages and inhibited IL-7 expression in macrophages, thereby alleviating the inflammatory response [53]. In addition, exosomes derived from different cells can regulate intestinal immunity by regulating the expression of inflammatory and anti-inflammatory factors. Dendritic cell (DC)-derived exosomes modulate the immune response and prevent the development of autoimmune diseases [54–56]. Wang et al. found that exosomes from Staphylococcus enterotoxin A (SEA)-treated DCs could regulate the production of inflammatory cytokines in mice with acute DSS-induced colitis, which could reduce the expression of the proinflammatory cytokines TNF-α, interferon (IFN)-γ, IL-17A, IL-12, and IL-22. The expression level of the anti-inflammatory cytokine TGF-β was increased, and the intestinal inflammatory response was alleviated [57]. Exosomes from hucMSCs reduced the expression of the proinflammatory cytokines TNF-α, IL-1β and IL-6 in the colon in IBD mice, increased the expression of the anti-inflammatory cytokine IL-10 and attenuated the inflammatory response, thereby alleviating DSS-induced colitis in mice [53]. M2 macrophage-derived exosomes upregulated Treg cells and IL-4, reduced the production of the proinflammatory cytokines IL-1β, IL-6 and IL-17A, reduced the severity of DSS-induced colitis in mice, and played a protective role in colitis [58]. Extracellular vesicles (EVs) secreted by hookworms could inhibit the production of key cytokines (IL-6, IL-1β, IFNγ and IL-17A) in the colon tissue of mice, while the anti-inflammatory cytokine IL-10 can reduced the intestinal inflammatory response [59]. Exosomes derived from bone marrow DCs treated with IL-10 inhibited the expression of the proinflammatory cytokines IL-2, IFN-γ and TNF-α and trinitrobenzene sulfonate-induced colitis [54]. Exosomes from different sources can reduce the severity of intestinal enteritis by regulating the expression of inflammation-related factors, providing a new perspective for the treatment of IBD, and the application of exosomes may become a new method for the treatment of IBD.

Exosomes not only participate in disease development but may also be used in disease diagnosis and prognosis. Wong et al. isolated exosomes from DSS-induced colitis mice models and used proteomics analysis to identify 56 differentially expressed proteins, most of which were acute phase proteins and immunoglobulins, suggesting that specific exosomal proteins are potential inflammatory markers of IBD [60]. According to the literature, oral lesions often occur concurrently with IBD and usually resolve when intestinal inflammation improves, suggesting that the occurrence of oral lesions is associated with the incidence of IBD. Salivary exosomes from IBD patients and healthy controls were examined, and the results showed that the expression of PSMA7 in the oral epithelium in the IBD group was higher, which was consistent with the expression of PSMA7 in colonic tissues in the IBD group. These results suggest that salivary exosomal PSMA7 is an important protein biomarker for IBD, which may be used as a diagnostic tool for this disease [61]. MiRNAs are small coding RNAs involved in the regulation of human gene expression. Exosomal miRNAs are differentially expressed in different diseases and can be used as a diagnostic markers. Wu et al. found that microRNAs were differentially expressed in UC and changed the expression of macrophage inflammatory peptide 2α [62]. A study performed miRNA microarray analysis of peripheral blood samples isolated from patients with CD) and UC and healthy controls, compared the miRNA expression patterns of exosomes between these three groups, and found that miRNA expression was different between IBD patients and healthy controls. Furthermore, miRNA expression was also different between CD patients and UC patients [63, 64]. This finding suggests that miRNAs in the blood may be useful diagnostic tools for IBD. However, the ability to distinguish IBD subtypes needs to be further studied.

### Exosomes and hepatitis

Hepatitis is a general term for inflammatory diseases of the liver, which are caused by a variety of pathogenic factors, such as viral infection, alcoholism, and autoimmune factors, and can be divided into acute and chronic hepatitis according to disease duration. Acute hepatitis is easily transformed into chronic hepatitis if not treated in time, while chronic hepatitis is often the basis for the occurrence of liver fibrosis and liver cancer [65]. Therefore, early detection and treatment of hepatitis is key to preventing further exacerbation and curing the disease. It has been reported that exosome-mediated cell communication plays an important role in the maintenance of physiological function and disease treatment. During viral infection, exosomes can spread the virus among cells without triggering immune recognition or being neutralized by antibodies in vivo. Exosomes play an important role in liver inflammatory diseases by participating in the pathogenesis and immune response of hepatitis and provide new ideas for the treatment of some hepatitis diseases, which has important clinical significance.

#### Exosomes and hepatitis A

Hepatitis A virus (HAV) infection is the main cause of hepatitis A. Studies have shown that the mechanism of HAV infection is related to exosomes. Ramakrishnaiah et al. reported that the HAV can hide in the exosomes membrane to escape the host immune response, survive in the blood, cause corresponding lesions, and possibly promote the spread of the virus in the liver [66]. Feng et al. found that HAV released from cells is masked by host-derived membranes, and viral particles can be engulfed by exosome-like host membranes to form enveloped viruses, thereby protecting virions from antibody-mediated neutralization [67]. However, viral exosomes or enveloped HAV particles (EHAVs) can also spread to plasmacytoid DCs (PDCs), inducing the activation of type I IFN and promoting the innate immune response [68]. These results suggest that viral exosomes can promote viral immune escape by masking viral particles, such as in the case of HAV infection, and can also activate PDCs and the innate immune response.

#### Exosomes and hepatitis B

**Fig. 3:**
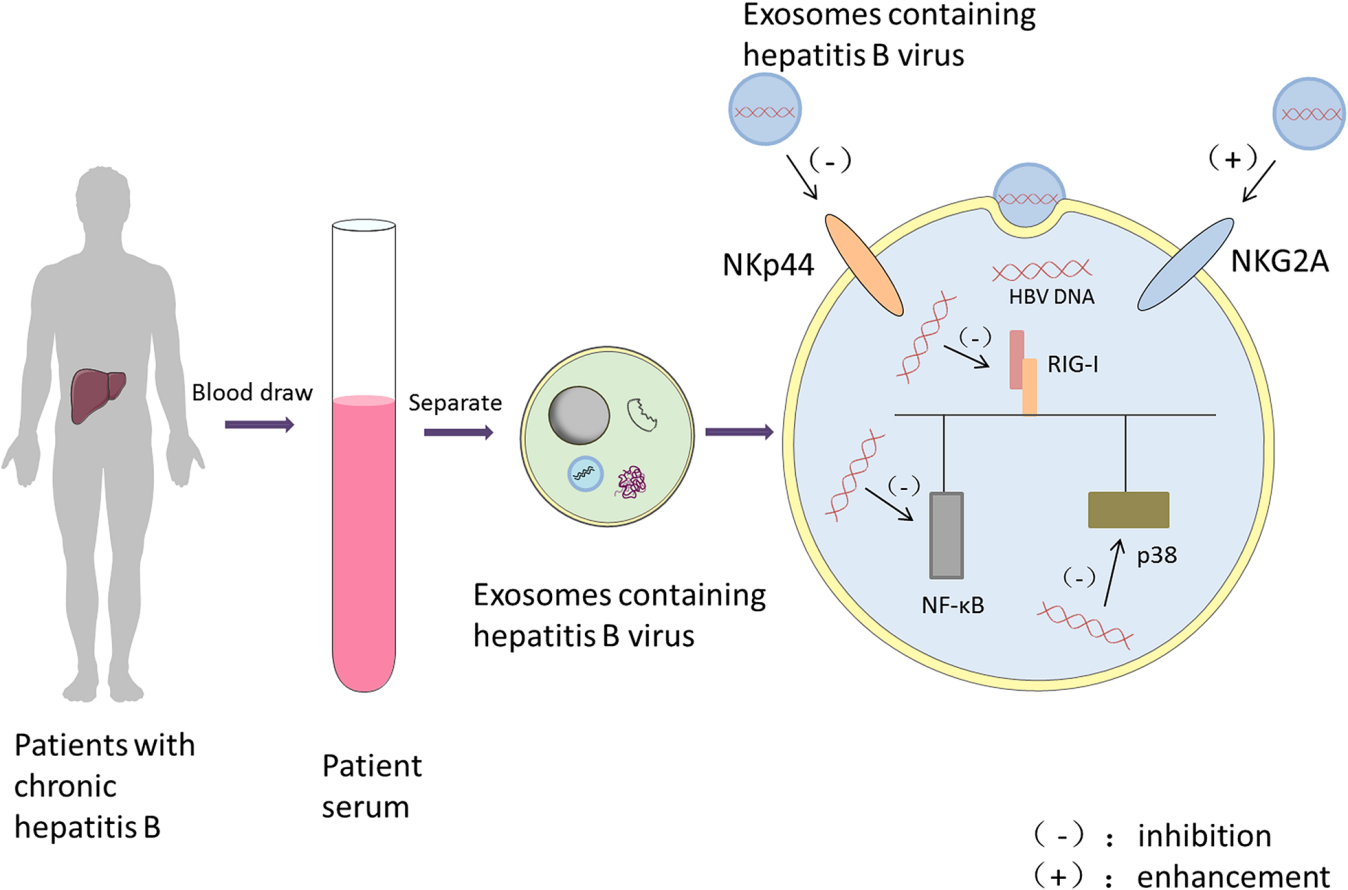
Exosomes are involved in the progression of hepatitis B. Exosomes circulating in the serum of patients with chronic hepatitis B contain HBV nucleic acids and proteins. Exosomes can reduce the expression of the activating receptor NKp44 on NK cells, enhance the expression of the inhibitory receptor NKG2A, and promote NK cell dysfunction. CHB exosomes can transfer HBV to NK cells, and HBV nucleic acids can downregulate the expression of RIG-I, inactivate the NF-κB and p38 pathways, induce functional tolerance of NK cells, and promote HBV viral replication

The extent of hepatitis B virus (HBV) damage is determined by the strength of the immune response and immune regulation. After HBV infection, the body produces a series of immune responses to fight the virus, but immunopathological liver damage can also occur. If the process continues, it will lead to chronic disease, resulting in chronic inflammatory changes in the liver. The mechanism of its pathogenesis is very complex. The immune function of the body is low. May mechanisms that cause the virus to escape the immune system may allow the virus to continue to replicate, thus producing a chronic infection. Exosomes have been reported to play an important role in the development of HBV infection. Natural killer (NK) cells control HBV replication in the early stage, but their antiviral activity is limited as chronic infection progresses [69]. Yang et al. found that serum exosomes of chronic hepatitis B (CHB) patients can reduce the expression of the activating receptor NKP44 on NK cells and enhance the expression of the inhibitory receptor NKG2A, promoting NK cell dysfunction [70]. In addition, CHB exosomes can transfer HBV into NK cells, and HBV nucleic acids can downregulate RIG-I expression and inactivate the NF-κB and p38 pathways, induce functional tolerance in NK cells and promote HBV viral replication (Fig. 3). Studies have shown that HBV infection of hepatocytes increases the production of exosomal miR-21 and other immunosuppressive miRNAs [71]. These exosomal miRNAs downregulate IL-12 expression, which may be the mechanism by which the virus escapes the host innate immune response. This finding suggests that exosomes play an important role in the maintenance of HBV infection in the host liver. Some viruses can use the exosome pathway for cell-to-cell transmission, thus avoiding surveillance by the immune system [72–74]. In addition, exosomes may facilitate the interaction between HBV-infected hepatocytes and other uninfected cells and host immune cells. HBV RNA and viral DNA were detected in CD81 + exosomes derived from HepG2 cells with pHBV, which can transmit HBV to uninfected cells. Circulating exosomes in the serum of patients with CHB contain HBV nucleic acids and HBV proteins. These exosomes modulate the innate immune response to HBV infection [71], and HBV-modified foreign bodies can transmit signals to immune cells and mediate cell–cell communication and immune regulation [75]. Exosomes secreted by hepatocytes contain many proteins that play important roles in the liver microenvironment. Several proteins were detected in HepAD38 (human liver cancer cell)-derived exosomes, such as HSP60, cell adhesion molecule 1, cluster proteins, the tyrosine protein kinase Lyn, and the receptor tyrosine protein phosphatase C. These proteins have been reported to be involved in the regulation of cytokine production or cytokine-mediated signalling pathways [76] and may help regulate immune responses during HBV infection. As mentioned previously, exosomes function as a double-edged sword during HBV infection. On the one hand, exosomes affect the immune response. On the other hand, exosomes can be an effective way to spread the virus. These findings provide new ideas for the interaction between HBV and the host and provide a basis for further research on the role of exosomes in HBV infection and pathogenesis.

Early studies have demonstrated that IFN-α is effective in treating HBV infection [77, 78]. Studies have shown that IFN-α induces an antiviral response in liver nonparenchymal cells and can be transported to infected hepatocytes via exosomes, thereby restoring the antiviral status of hepatocytes [79, 80]. This finding suggests that exosomes can act as vectors and promote the antiviral effects of IFN-α. However, for some patients with CHB, IFNα is less effective, and the mechanism is related to exosome-mediated IFITM2. Exosomes mediate the transport of IFITM2 from hepatocytes to DCs, enhance IFITM2-mediated inhibition of endogenous IFN-α synthesis, and inhibit the anti-HBV efficacy of exogenous IFN-α, resulting in poor efficacy in CHB patients with exogenous IFN-α therapy [81]. Therefore, further study of exosomes may be a potential therapeutic strategy for HBV.

#### Exosomes and hepatitis C

Hepatitis C is caused by hepatitis C virus (HCV) infection. This condition easily progresses to a chronic disease and further develops into cirrhosis, liver failure and hepatocellular carcinoma. Hepatitis C seriously affects quality of life and increases socioeconomic burdens [82]. Studies have shown that exosomes can mediate the cell-to-cell spread of HCV, serve as a potential immune escape or response mechanism, and promote the persistence of the virus [83]. Exosomes in plasma from patients with hepatitis C contain HCV RNA [84, 85] and can form protein complexes with Ago2, HSP90 and miR-122 to enhance the stability and infectivity of viruses and promote their replication and transmission [86–88]. Early studies have shown that HCV infection results in the expansion of T follicular regulatory (TFR) cells, which are increased in the liver during chronic HCV infection [89]. Recent studies [90, 91] have shown that exosomes isolated from the plasma of HCV patients and the supernatant of HCV-infected liver cells can promote the differentiation of TFR cells by inhibiting the amplification of myeloid-derived inhibitory cells (MDSCs) by inhibiting miR-124. Exosomes also act directly on CD4 + T cells, promoting TFR cell development and TFR cell amplification in a TGF-β-dependent manner and enhancing the TFR cell response, thereby inhibiting the effects of follicular helper T (TFH) cells and B cells, inhibiting the production of autoantibodies, and leading to viral persistence. HCV-related exosomes are also involved in immune escape. Exosomes derived from HCV-infected cells can stimulate monocytes to secrete hemicylocytin 9 (Gal-9), which can inhibit the specific immune response mediated by T cells after binding with T cell immunoglobulin mucin molecule 3 (Tim-3), leading to immune dysfunction [92, 93]. A study also identified exosomes containing HCV RNA, which could effectively transmit HPV in vitro and resist the effects of anti-HCV neutralizing antibodies [82]. In addition, exosomes have been reported to be involved in inhibiting viral replication. HCV RNA in hepatocellular-derived exosomes from HCV-infected patients can be transferred to PDCs to trigger IFN-α production [94]. Qian et al. reported that exosomes secreted by hucMSCs inhibited HCV infection in vitro, and hucMSC-derived exosome therapy showed synergistic effects when used in combination with IFN-α or trapvir, which was approved by the US Food and Drug Administration, enhancing the anti-HCV effect [95]. These results demonstrate the mechanism of exosomes in HCV infection, explain viral immune escape, and provide novel insights into the transmission and pathogenesis of this disease. Enhancing our understanding of viral persistence in human chronic viral infections will provide novel insights into the transmission and pathogenesis of HCV, and the prospects for HCV treatment.

#### Exosomes and autoimmune hepatitis

Autoimmune hepatitis is a chronic progressive inflammatory liver disease mediated by the autoimmune response. IL-1β and IL-6 participate in the occurrence and development of autoimmune hepatitis [96]. STAT3 is an important activator of IL-1β and IL-6 [97]. Lu et al. found [98] that MSC-derived exosomes can transmit miRNA-223-3p to inhibit the expression of the inflammation-related gene STAT3, thus regulating the expression of IL-1β and IL-6 in the liver, changing the proportion of Treg and Th17 cells in mice, increasing the Treg/Th17 cell ratio, reducing inflammatory reactions and reducing liver injury. In mice treated with bone marrow-derived mesenchymal stem cell (BMSC)-derived exosomal miR-223 ( +) for autoimmune hepatitis, significant reductions in ALT and AST and proinflammatory cytokines were observed [99]. These findings suggest that BMSC-derived exosomal miR-223 may be a potential treatment for autoimmune hepatitis, providing a new therapeutic option for patients with autoimmune hepatitis.

### Exosomes and pancreatitis

Pancreatitis is inflammation of the pancreas, and includes acute and chronic pancreatitis. Acute pancreatitis (AP) is an inflammatory injury characterized by pancreatic oedema, bleeding and necrosis caused by the destruction of pancreatic tissue due to a variety of causes. During disease progression, multiple organ dysfunction and local complications of the pancreas may occur. Treatment mainly involves identifying the cause and controlling inflammation, but there is poor prognosis and high mortality [100, 101]. If the aetiology is not removed, AP may occur repeatedly, and repeated inflammation and fibrosis may develop into chronic pancreatitis, which leads to irreversible damage to pancreatic function and is one of the risk factors for pancreatic cancer [102, 103]. Therefore, it is very important to understand the pathogenesis of pancreatitis and intervene as soon as possible. Exosomes mediate antigen presentation and carry different proteins and miRNAs involved in the regulation of pancreatitis.

Research [104] has shown that during AP, two distinct exosome populations are produced: plasma exosomes and pancreatitis-associated ascites (PAAF) exosomes. Their origin, tissue distribution, molecular contents and physiological effects are significantly different. During AP, the proinflammatory effects of plasma exosomes on macrophages is higher than that of PAAF exosomes, which can promote the development of systemic inflammation. In AP, inflammatory mediators are increased, leading to a large number of inflammatory exudations, which in turn leads to systemic inflammation, inflammatory injury and dysfunction in multiple organs. Macrophages play an important role in the systemic inflammatory response caused by AP, and modulating macrophage activation during AP can reduce the inflammatory response. Studies have shown that activated pancreatic acinar cell-derived exosomes and exosomal RNAs are involved in regulating macrophage activation and inducing the inflammatory response [105]. NLRP3 inflammasome activation and alveolar macrophage (AM) activation are the causes of secondary lung injury in AP. Plasma-derived exosomes from AP mice trigger NLRP3 inflammasome activation and induce AM activation. By inhibiting the release or uptake of exosomes in vivo, AP-induced lung injury can be alleviated. Exosomes in lung inflammation associated with experimental AP were studied, and the results showed [106] that the number and concentration of circulating exosomes in the blood of rats increased significantly during AP. These exosomes reached the alveolar space and activated AMs into the proinflammatory M1 phenotype, promoting the pulmonary inflammatory response. These exosomes can also cross the alveolar epithelial barrier and be engulfed by AMs, which can exacerbate inflammation. These results suggest that exosomes play an important role in the mechanism of lung injury in AP, have proinflammatory properties and mediate disease development.

The immunosuppressive and anti-inflammatory properties of MSCs are ideal for the treatment of pancreatitis [107, 108]. Studies have reported that exosomes can be used as drug carriers for the treatment of diseases. Exosomes derived from MSCs have therapeutic effects on various physiological and pathological conditions. In one study, it was found that [109] klotho in MSC-derived exosomes modulated inflammation and apoptosis during AP. By inhibiting NF-κB signalling, the release of IL-6 and TNF-α in an animal model of AP was reduced, exerting an anti-inflammatory effect. Exosomes not only participate in the pathogenesis of AP and its complications but also play a role in its treatment, which provides new strategies to treat AP. These findings suggest that exosomes may be potential targets for controlling the systemic inflammatory response during AP and have important implications for future studies of exosomes and pancreatitis.

Acute pancreatic recurrence easily leads to chronic progressive inflammation, and pancreatic tissue continues to develop irreversible lesions and even cancer, which seriously affects quality of life. Pancreatic injury due to pancreatitis is associated with fibrosis due to the production of CCN2-dependent collagen by pancreatic stellate cells (PSCs). Charrier et al. found that [110] connective tissue growth factor regulated resting PSCs and changed their phenotype and function, and these cells became active PSCs. The increased expression of miR-21 in activated PSCs can promote the expression of CCN2, while the increase in CCN2 can also stimulate the expression of miR-21. Therefore, the miR-21-CCN2 axis in activated PSCs forms a positive feedback loop, interacting with each other and leading to tissue fibrosis. In addition, CCN2 or miR-21 in PSC-derived foreign bodies was absorbed by other PSCs to stimulate fibrosis, suggesting that exosomes can mediate fibrotic signal transduction in PSCs, which is how PSC-derived exosomes regulate pancreatic fibrosis and affect the progression of chronic pancreatitis. The exosomal miR-21-CCN2 axis is a novel pathway associated with fibrotic signal transduction in PSCs. Activation of PSCs is a key step in pancreatic fibrosis [111]. Activated PSC-derived exosomes play an important role in the progression of chronic pancreatitis. Blocking specific exosome-mediated pathways of information and substance exchange can block the progression of chronic pancreatitis. Inhibiting PSC activation may also be a treatment strategy for chronic pancreatitis. More in-depth research is expected to provide new methods and strategies for the clinical treatment of chronic pancreatitis.

## Summary and outlook

Exosomes are important carriers of signals and substances between cells. They plays important roles in the occurrence and development of inflammatory diseases in the digestive system, participate in the expression of inflammatory factors and modulate the inflammatory response, providing new perspectives for the treatment of diseases. Furthermore, specific miRNAs or proteins in exosomes may be potential biomarkers for inflammatory diseases of the digestive system and have diagnostic potential. Furthermore, in viral hepatitis, exosomes can encapsulate viral particles, evade the host immune response, promote viral transmission, lead to chronic inflammation, and act as targets of immunotherapy. However, although progress has been made on research on exosomes in inflammatory diseases of the digestive system and exosomes have some potential in the diagnosis and treatment of diseases, there are still challenges in their clinical application. To better use exosomes in clinical applications, we need to perform a large number of clinical trials to fully understand the function and mechanism of exosomes so that their unique advantages can be used in the clinical diagnosis, prevention and treatment of diseases.

**Table 1:** Exosomal components play different roles in inflammatory diseases of the digestive system

Source	Exosomal composition	Disease	Result	The role of components in exosomes	References
Rat serum	Serum exosomal miR-29a-3p	Reflux esophagitis	Increased expression	A specific marker for the diagnosis of chronic RE	[40]
Tongue-exfoliated cells	Exosomal miR-203 in tongue exfoliated cells	Gastroesophageal reflux disease	Decreased expression	A marker for the diagnosis of GERD	[41]
HP-infected Macrophages	miR-155 in exosomes of HP-infected macrophages	Chronic gastritis caused by HP infection	Increased expression	Regulate inflammation	[43]
Serum of patients with CAG	hsa-miR-122-5p in serum exosomes	Chronic atrophic gastritis	Increased expression	Potential biomarkers for diagnosis of CAG	[47]
Saliva of IBD mice	salivary exosome PSMA7	Inflammatory bowel disease	Increased expression	Important protein biomarkers for the diagnosis of IBD	[61]
Hepatocytes	Exosomal miR-21	Chronic hepatitis B	Decreased expression of IL-12	Participate in virus escape	[71]
HepAD38 cells	Proteins in exosomes: HSP60 and cell adhesion molecules	Hepatitis B	Regulation of signalling pathways	Regulate the immune response	[76]
BMSCs	BMSC-derived exosomal miR-223	Autoimmune hepatitis	Inhibit the expression of proinflammatory factors	Treatment	[99]
MSCs	Klotho	Acute pancreatitis	Inhibit NF-κB signalling	Inhibit inflammation	[109]

## Data Availability

Not applicable.
